# Development of the RP-HPLC Method for Simultaneous Determination and Quantification of Artemether and Lumefantrine in Fixed-Dose Combination Pharmaceutical Dosage Forms

**DOI:** 10.1155/2024/3212298

**Published:** 2024-02-07

**Authors:** Simon Nyarko, Kwabena Ofori-Kwakye, Raphael Johnson, Noble Kuntworbe, Desmond Asamoah Bruce Otu, Denis Dekugmen Yar, Yaa Asantewaa Osei

**Affiliations:** ^1^Department of Pharmaceutics, Kwame Nkrumah University of Science and Technology, Kumasi, Ghana; ^2^Department of Public Health, Akenten Appiah-Menka University of Skills Training and Entrepreneurial Development, Mampong, Ghana

## Abstract

Developing countries face enormous challenges with substandard and falsified antimalarial drugs. One specific issue is the lack of a simple, cost-effective, and robust HPLC method to simultaneously determine and quantify the active pharmaceutical ingredients (APIs) in fixed-dose artemether-lumefantrine pharmaceutical dosage forms. The current study developed a novel, simple, sensitive, precise, accurate, and cost-effective RP-HPLC method for the simultaneous determination and quantification of artemether and lumefantrine in pharmaceutical dosage forms. The HPLC analysis was carried out on an Agilent 1260 Infinity Series HPLC system equipped with an ODS Intersil-C8 (150 × 4.6 mm) 5.0 *µ*m column, by isocratic elution. The mobile phase composition consisted of acetonitrile and 0.05% orthophosphoric acid buffer of pH 3.5 in the ratio of 70 : 30 v/v. The analysis was performed at a 1 mL/min flow rate and a column temperature of 25°C. The total run time was 6 minutes. The detection was done with a variable wavelength detector (VWD) at an isosbestic point wavelength (*λ*) of 210 nm. The developed method was validated according to the ICH guidelines concerning system suitability, specificity, linearity, accuracy, precision, and robustness. The system suitability of the developed method revealed satisfactory theoretical plates and symmetry factors. The method proved to be specific, with no interference of mobile phase or excipients. The calibration plot exhibited linearity over the concentration range of 275–1925 *μ*g/mL with *R*^2^ = 0.9992 for artemether and a range of 150–1050 *μ*g/mL with *R*^2^ = 0.9985 for lumefantrine. The accuracy of the method, determined by the recovery study, was 99.79–100.16% for artemether and 99.04–99.50% for lumefantrine. The % RSD values for intraday precision were 0.175 and 0.203, while interday precision values were 0.340 and 0.554 for artemether and lumefantrine, respectively. The method demonstrated robustness when subjected to slight modifications in the flow rate, column temperature, and mobile phase composition. The developed analytical method proved satisfactory as per ICH guidelines and hence can be used for the determination and quantification of artemether and lumefantrine in bulk drug and pharmaceutical dosage forms.

## 1. Introduction

At present, artemisinin-based combination therapy (ACT) is regarded as the most rapid and effective antimalarial treatment available. Malaria is a common fever disease that frequently necessitates hospitalization after travelling to areas where it is endemic [1]. Following the WHO recommendations, Ghana moved from chloroquine to artemisinin-based combination therapy (ACT) as the first-line treatment for uncomplicated malaria in 2005. Since 2014, Ghana has made extensive use of three ACTs, including dihydroartemisinin–piperaquine (DHAP), artemether–lumefantrine (AL), and artesunate–amodiaquine (AA). Fixed-dose combination (FDC) artemether–lumefantrine (AL) drug is authorized for the treatment of malaria caused by *Plasmodium falciparum* [2]. AL is an ACT made of two active ingredients, namely, artemether, which is derived from the plant *Artemisia annua*, and lumefantrine, which is synthesized chemically [3]. Together, these two compounds work to inhibit the growth and replication of the malaria parasite, thus curing the infection. Although the preferred treatments for severe malaria are intravenous/intramuscular (IV/IM) artesunate, IV/IM quinine, and IM artemether [4]; tablets and suspensions are considered the most widely used dosage forms of fixed-dose combination artemether–lumefantrine drug available in the market.

At the moment, poor-quality medicines present tremendous hurdles for poor nations [5] due to the lack of a simple, less expensive, robust, and stable HPLC method that could simultaneously determine the content of the APIs in artemether–lumefantrine tablets and suspensions in resource-limited settings. There is, therefore, an urgent need for methods that are accurate, cost-effective, easy to use, and rapid and require the use of uncomplicated equipment to facilitate easy identification and quantification of the active components in fixed-dose combinations of artemether and lumefantrine dosage forms for in-process quality control checks or routine pharmacovigilance [6].

High-pressure liquid chromatography (HPLC) is a widely used analytical technique for the separation, identification, and quantification of drugs in pharmaceutical formulations. It is a highly sensitive and selective method that can detect and quantify trace amounts of drugs [7]. However, developing an HPLC method for the simultaneous determination of artemether and lumefantrine in FDC has proven to be difficult due to the physicochemical properties of the drugs [8].

**Figure 1:**
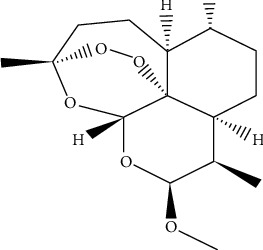
Artemether chemical structure.

**Figure 2:**
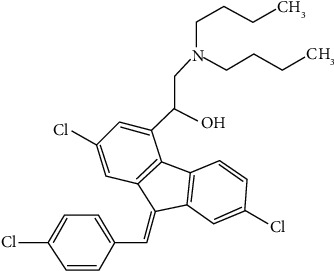
Lumefantrine chemical structure.

Artemether is a form of artemisinin in which the lactone has undergone conversion to the associated lactol methyl ether [9]. It is insoluble in water but soluble in oil. Lumefantrine belongs to the class of fluorenes and is composed of the compound 9-(p-chlorobenzylidene)-9H-fluorene, which has chlorine substituents at positions 2 and 7 and a 2-(dibutylamino)-1-hydroxyethyl group at position 4 [10]. Figures 1 and 2 illustrate the chemical structure of artemether and lumefantrine, respectively.

Many studies have developed methods to quantify the API content in drugs, with the majority being used for tablet formulations. Most of these methods are not able to determine simultaneously the active ingredients present in fixed-dose combination medicines [11]. Due to their distinct chemical properties, including factors such as polarity, solubility, and UV absorption, the APIs artemether and lumefantrine (classified as BCS class II or IV drugs) exhibit poor water solubility and limited permeability [12]. These differences make it challenging to develop an HPLC method that can separate and quantify both drugs simultaneously in the same sample. In addition, artemether and lumefantrine have different retention times in HPLC columns, which further complicate the simultaneous determination of both drugs. Numerous endeavors have been undertaken to establish high-performance liquid chromatography (HPLC) methodologies for the concurrent quantification of artemether and lumefantrine in fixed-dose combination (FDC) drugs. Regrettably, many of these methods exhibit deficiencies in sensitivity, selectivity, precision, and accuracy. Notably, the prevalent issue persists where, even within a fixed-dose combination drug, the analysis of each active pharmaceutical ingredient (API) in pharmaceutical and biological matrices necessitates separate procedures. Other challenging issues are the cost of analytical reagents used and the long run time. A study by Shamshad et al. [13] utilized a high injection volume of 20 *µ*L, making the method less economical. Another study by Debrah and colleagues [14] was done at a temperature of 40°C. A high column temperature makes the method unfavorable for commercial use.

This study aimed to develop and validate a novel reverse-phase high-performance liquid chromatography (RP-HPLC) method capable of concurrently detecting artemether and lumefantrine within a single solvent system within a single short run. This has the potential to significantly reduce analysis time for fixed-dose combination artemether and lumefantrine pharmaceutical dosage forms, consequently minimizing overall costs associated with the analytical process and thus underscoring the necessity of the study.

## 2. Materials and Methods

### 2.1. Chemicals and Analytical Reference Standards

Primary analytical reference standards of purity ≥98% artemether and lumefantrine (Sigma-Aldrich, USA), deionized water (gifted by Tradewinds Chemist Limited, Ghana), analytical grade solvents including HPLC grade acetonitrile of purity ≥99.9% (LiChrosolv® Reag. Ph Eur. Supelco, Germany), and analytical grade orthophosphoric acid 85% (Supelco, Germany) were used.

### 2.2. RP-HPLC Method Development

#### 2.2.1. Instrumentation and Analytical Conditions

The HPLC analyses were carried out on an Agilent 1260 Infinity Series HPLC system (Agilent Technologies, Santa Clara, California, USA), equipped with a quaternary pump, autosampler, variable wavelength detector (VWD), and an ODS Intersil-C8 column (Phenomenex) (150 × 4.6 mm, 5.0 mm particles). The HPLC was controlled by a PC workstation (HP) using ChemStation software. All mobile phase solutions were degassed ultrasonically using a sonicator (Fisherbrand, model no. FB15053) before use.

#### 2.2.2. Chromatographic Parameters

Thorough investigations and assessments of the physicochemical characteristics of each active pharmaceutical ingredient (API) were conducted before the HPLC method development process. The powder of the various APIs was subjected to thorough testing to evaluate various qualities such as solubility, polarity, purity, and maximum absorption wavelength. Through extensive testing and careful analysis, key technical parameters for the method were determined, including the appropriate stationary phase, mobile phase, elution type, and common wavelength for detecting both APIs. Given the need to achieve complete dissolution of all APIs, combinations of solvent systems, such as acetonitrile, methanol, water, and acetone, were examined. After a thorough evaluation, the most suitable solvent was chosen for the subsequent validation process.

#### 2.2.3. Analytical Conditions

The column temperature, flow rate, injection volume, and run time were 25°C, 1.0 mL/min, 5.0 *μ*l, and 6 min, respectively. UV detection was performed at 210 nm.

#### 2.2.4. Preparation of 0.05% Orthophosphoric Acid

A solution of 0.05% orthophosphoric acid was prepared by dissolving 0.5 mL of the acid in deionized water. The total volume was then brought up to 1000 mL and filtered through grade 2 qualitative filter paper (Whatman®) with an 8 *μ*m pore size.

#### 2.2.5. Preparation of Diluent

The diluent was made of acetonitrile and 0.05% orthophosphoric acid (70 : 30 v/v) prepared in separate flasks for dissolving both the standard and samples.

#### 2.2.6. Solution Stability Study

A stability study was conducted on the solution containing active pharmaceutical ingredients (APIs) and the diluent to evaluate its ability to maintain chemical and physical integrity under defined storage conditions and over a specified period. Throughout the study, the concentration of both artemether and lumefantrine exhibited a change of less than 1% from the initial value over a 24-hour storage period at room temperature (25°C).

#### 2.2.7. Filter Compatibility Studies

Filter compatibility studies were conducted using Whatman® grade 1 and grade 2 qualitative filter papers with pore sizes of 11 *μ*m and 8 *μ*m, respectively. Evaluation of drug release in standard and sample solutions indicated that both filter papers demonstrated excellent compatibility, with a relative standard deviation of less than 2%. This suggested that both grade 1 and grade 2 filter papers were suitable for the intended purposes. Following this, the grade 2 qualitative filter paper with a pore size of 8 *μ*m was chosen for all subsequent filtrations.

#### 2.2.8. Preparation of the Mobile Phase

An isocratic mobile phase containing acetonitrile and 0.05% orthophosphoric acid (buffer) in the ratio 70 : 30 (v/v) was sonicated and filtered through grade 2 qualitative filter paper (Whatman™) of pore size 8 *μ*m and used at a flow rate of 1.0 mL/min. The separation of artemether and lumefantrine was evaluated in different proportions of these solvents and, for each condition, the retention factor (*k*) and resolution (*R*) were determined.

#### 2.2.9. Wavelength and the Flow Rate

Employing a high-performance liquid chromatography (HPLC) instrument, a comprehensive scan was conducted over a wavelength range spanning from 200 nm to 400 nm, ensuring a thorough examination of absorption characteristics. The subsequent narrowing down of this spectrum to the selection of 210 nm yielded an isosbestic point, which proved advantageous for both APIs.

In tandem with the spectral optimization, adjustments were made to the pump system to regulate the delivery of the mobile phase at varying flow rates, namely, 0.5, 0.6, 0.8, 0.9, 1.0, 1.10, 1.5, and 2.0 ml/min. Subsequent analysis of all experimental runs led to the determination that a flow rate of 1 ml/min was the optimal choice for method development. This particular flow rate was chosen for its demonstrated ability to facilitate efficient separations, maintain reasonable retention times, and enhance the resolution of chromatographic peaks when compared to the other tested flow rates.

#### 2.2.10. Preparation of Standards

HPLC grade reference standards equivalent to 30 mg and 55 mg of lumefantrine and artemether, respectively, were transferred into a 50 mL clean dry volumetric flask. Acetonitrile was added first to the powdered mixture and sonicated for 10 minutes and then topped up with 0.05% orthophosphoric acid to make up the volume of 50 mL in an equivalent ratio of 70 : 30 (v/v). The resulting mixture was finally sonicated for better dissolution and filtered via grade 2 qualitative filter paper (Whatman®) of pore size 8 *μ*m into vials.

#### 2.2.11. Preparation of Tablet and Suspension Samples

In this study, six tablets of artemether–lumefantrine (AL) were analyzed for their average weight. The tablets were then crushed into a fine powder and a specific amount of this powder, equivalent to 30 mg of lumefantrine and 55 mg of artemether, was placed in a 50 mL flask. To prepare the analytical solution, acetonitrile was added to the powder and sonicated for 10 minutes. This mixture was then combined with a 0.05% orthophosphoric acid solution in a ratio of 70 : 30 v/v, resulting in a final volume of 50 mL. The mixture was further sonicated for better dissolution and was filtered through 8 *μ*m pore size grade 2 qualitative filter paper (Whatman®). The vials used for filtration were thoroughly cleaned, rinsed, and dried beforehand.

The suspensions were reconstituted according to the specifications given by the manufacturer. A quantity of the suspension sample equivalent to 30 mg of lumefantrine and 55 mg of artemether was accurately measured in a 50 ml volumetric flask. Acetonitrile was added first to the powdered mixture and sonicated for 10 minutes and then topped up with the buffer (0.05% orthophosphoric acid) to make up the volume of 50 ml in an equivalent ratio of 70 : 30 (v/v). The resulting mixture was finally sonicated for better dissolution and filtered via grade 2 qualitative filter paper (Whatman®) of pore size 8 *μ*m into vials that have been washed, rinsed, and dried completely.

### 2.3. Validation of the Method

Multiple metrics, including linearity, precision, accuracy, robustness, limit of detection, and limit of quantitation, were evaluated as part of the validation process [15]. To ensure conformity to globally accepted standards, the chromatographic method's validation was carried out following a research validation protocol inspired by the International Council for Harmonization of Technical Requirements for Pharmaceuticals for Human Use (ICH Q2b) [16–18].

#### 2.3.1. System Suitability

System suitability parameters, such as the theoretical plate, the tailing factor, the peak-to-noise ratio, the similarity factor, and the retention time, were carefully examined to make sure they complied with the acceptable ranges outlined in the guidelines provided by the European Pharmacopoeia and the Center for Drug Evaluation and Research (CDER). The peak area, retention time, and symmetry factor were calculated and analyzed for sextuplicate injections, and the symmetry factor was carefully scrutinized. Furthermore, theoretical plates that indicate column efficiency were analyzed.

#### 2.3.2. Specificity

High-performance liquid chromatography (HPLC) relies heavily on specificity since it allows the analytical technique to identify and separate the analyte from other components in a mixture. A series of injections were carried out using several solutions that each contained 5 *µ*L of the standard solutions of each API, a mixture of the APIs, an AL drug sample, and the blank (diluent) to evaluate the method's specificity. The subsequent analysis entailed scrutinizing the chromatographic profile for the presence of any interfering peaks that may compromise the accurate determination of the analyte of interest.

#### 2.3.3. Linearity

The exact weights of each sample were accurately measured and then diluted to a fixed volume of 50 mL using the mobile phase as the diluent, resulting in the creation of nine (9) distinct concentration levels. Standard solutions encompassing concentrations ranging from 275 *μ*g/mL to 1925 *μ*g/mL of artemether and from 150 *μ*g/mL to 1050 *μ*g/mL of lumefantrine were prepared and injected. For each concentration level, a consistent injection volume of five microliters (5.0 *µ*L) was used. The resulting chromatographic data were graphically plotted against their corresponding concentrations. The obtained *R*^2^ values demonstrated a linear correlation between peak areas and the concentrations of the reference solutions, affirming the method's linearity. Furthermore, rigorous statistical analyses were conducted to validate the linearity of this novel method.

#### 2.3.4. Calibration Curve for Artemether and Lumefantrine

To establish the relationship between the concentrations of the solutions and the corresponding peak areas, the mean of the peak areas was calculated and plotted against their respective concentrations. By plotting these data points, distinct calibration curves for artemether and lumefantrine were generated. These calibration curves serve as references to estimate the amount of the respective APIs present in the samples.

#### 2.3.5. Precision

Repeatability and intermediate precisions were identified and statistically evaluated to establish the precision of the approach proposed.

*(1) Repeatability Precision*. The intraday precision was analyzed using sextuplicate of the standard solutions containing 0.800 mg/mL (80%), 1.100 mg/mL (100%), and 1.320 mg/mL (120%) of the stock solution. The mean chromatogram peak areas of each standard solution of various concentrations were collected, and the relative standard deviation (RSD) was calculated.

*(2) Intermediate Precision*. The interday precision is useful in the validation process as it gives the precision of the results of the HPLC when run on different days or by different analysts using the same developed method. Sextuplicate of the standard solutions containing 0.800 mg/mL (80%), 1.100 mg/mL (100%), and 1.320 mg/mL (120%) of the stock solution was run on three different days. The mean chromatogram peak areas of each standard solution of various concentrations were collected, and the relative standard deviation (RSD) was calculated.

#### 2.3.6. Sensitivity

The limit of detection (LOD) and limit of quantification (LOQ) of the developed method were, respectively, calculated from the standard deviation and slope obtained from the calibration curve and the linearity test based on the signal-to-noise (S/N) ratio of 3 : 1 and 10 : 1 in the following formula:

(1) $$\begin{array}{c} \text{LOD}=3.3\times \left(\frac{SD}{S}\right), \end{array}$$

(2) $$\begin{array}{c} \text{LOQ}=10\times \left(\frac{SD}{S}\right), \end{array}$$

where *SD* = the standard deviation. *S* = slope of the calibration curve.

#### 2.3.7. Accuracy

In this study, to determine the accuracy of the proposed analytical method and detect any interference matrix from excipients used in the dosage forms, recovery experiments were conducted using the standard addition method (spiking). This method involved adding a known concentration of a standard to a fixed concentration of the preanalyzed sample solution. The percent recovery was then calculated by comparing the area before and after the addition of the working standard. Recovery of the method was carried out in three sets at different concentration levels corresponding to 80%, 100%, and 120% of the expected concentration. By comparing the amount claimed and the amount recovered after the addition of the standard, the percentage recovery was determined and % RSD was calculated.

#### 2.3.8. Robustness

A few chromatographic parameters were intentionally altered to test the robustness of the established approach. Specifically, the flow rate, column temperature, and mobile phase composition ratio were altered. The analysis was done by generating six sample solutions at 100% concentration. Sample solutions were injected, and the chromatographic parameters indicated above were modified in accordance to test the robustness of the devised method.

## 3. Results

### 3.1. Method Development

To optimize the separation of analytes of interest, extensive investigations were conducted on various chromatographic conditions. Parameters such as the mobile phase composition, stationary phase, column temperature, flow rate, wavelength, and injection volume were carefully studied. Through a series of rigorous trials, a reverse-phase, ODS Intersil-C8 column (Phenomenex) measuring 150 × 4.6 mm with 5.0 *μ*m particles was determined to be the most suitable stationary phase for the analysis. The mobile phase composition of acetonitrile and 0.05% orthophosphoric acid in a ratio of 70 : 30 (v/v) was found to provide optimal separation. Moreover, a detection wavelength of 210 nm was selected as the isosbestic wavelength, allowing for maximum sensitivity and selectivity. To maintain consistent and reproducible results, a column temperature of 25°C was deemed appropriate for the analysis. The flow rate of 1.0 mL/min was identified as the optimal rate, striking a balance between separation efficiency and analysis time. A minimal injection volume of 5.0 *μ*L was determined to be sufficient for achieving well-defined peaks and maintaining sensitivity.

Utilizing an isocratic elution method, the developed chromatographic conditions were further refined to yield well-distinguished peaks with consistent retention times. The entire analysis process was completed within a run time of 6 minutes.

### 3.2. Validation of the Method

#### 3.2.1. System Suitability

System suitability testing was performed to assess the suitability of the developed chromatographic system for analysis. Six replicate solutions of artemether and lumefantrine standards were utilized to evaluate retention times, symmetry factors, and theoretical plates. The obtained results indicate favorable system performance.

The peak symmetry exhibited satisfactory values, with an average of 0.91 for artemether and 1.32 for lumefantrine. The average retention times were determined as 5.38 minutes for artemether and 1.42 minutes for lumefantrine. In addition, the resolution was 2.03, and average theoretical plates were found to be 2297 for artemether and 5035 for lumefantrine, signifying a sufficient level of chromatographic resolution. The assessed parameters, including % RSD (≤2%) of peak areas and retention time, tailing factor (≤2), theoretical plates (≥2000), and peak resolution (*R*) (≥2), remained within the acceptable limits outlined by the British Pharmacopoeia (BP) requirements. Thus, the system suitability data, presented in Table 1, affirm that the developed chromatographic system meets the necessary criteria for the intended analysis.

#### 3.2.2. Specificity

**Figure 3:**
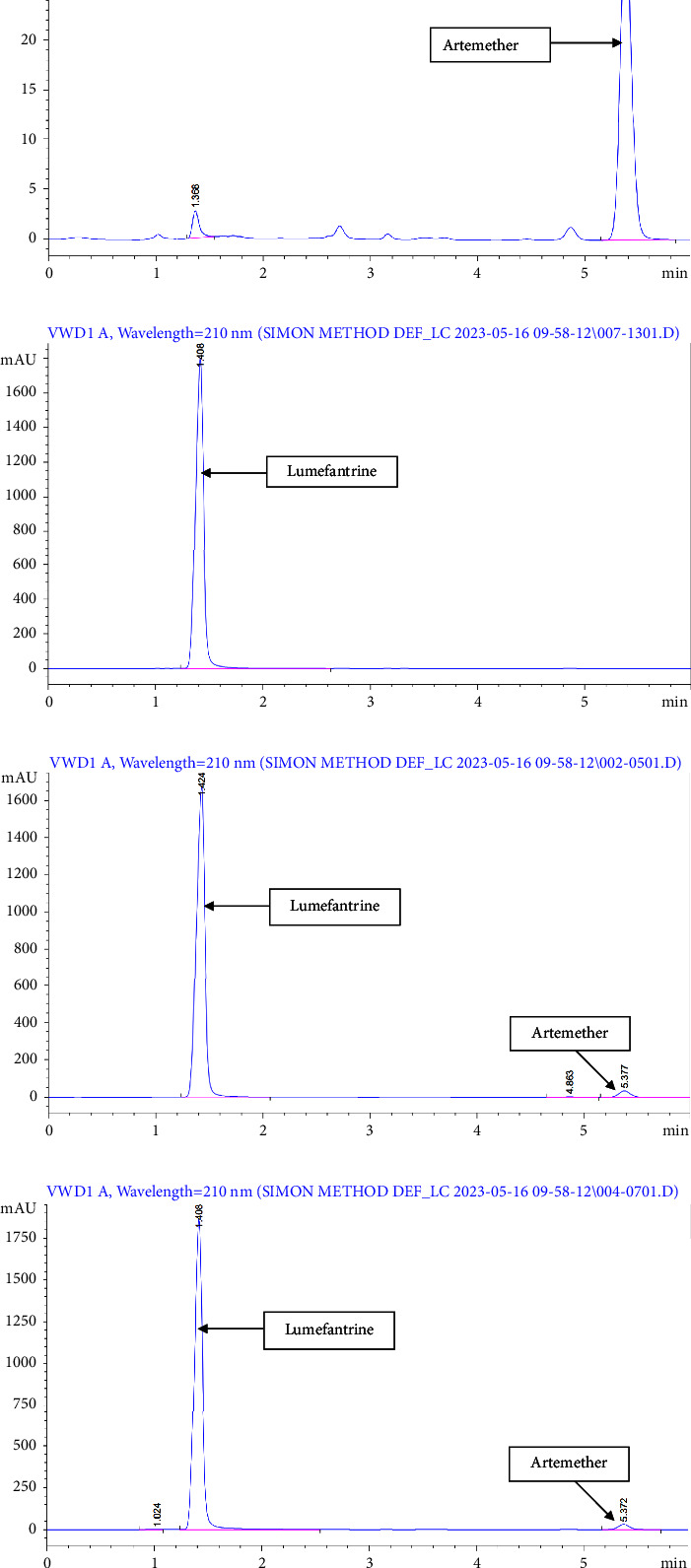
Chromatograms of (a) artemether standard (other smaller peaks observed are unknown impurities), (b) lumefantrine standard, (c) artemether–lumefantrine standard, and (d) artemether–lumefantrine sample.

The developed method exhibited excellent specificity for the resolution of all active pharmaceutical ingredients (APIs) used in the study. No interference peaks were observed during the elution periods of the APIs, even in the presence of excipients. The chromatograms in Figure 3 illustrate the method's specificity. These chromatograms provide visual evidence of the distinct separation of the APIs, without any overlapping or interfering peaks. The absence of extraneous signals within the elution regions of interest confirms the high specificity of the method.

#### 3.2.3. Linearity

A graphical representation of the analytical curve displaying the peak area against concentration was generated, and the result was subjected to regression analysis. From the regression analysis, the linear equation was obtained as follows: *y* = 0.2296*x* − 1.529 for artemether and *y* = 15.609*x* + 167.14 for lumefantrine, and their coefficient of determination (*R*^2^) was 0.9992 and 0.9985, respectively. The calibration curve of artemether and lumefantrine at 210 nm is shown in Figures I and II (supplementary materials), respectively, and the linearity results of artemether and lumefantrine are shown in Table 2.

#### 3.2.4. Sensitivity

The LOD signifies the lowest concentration at which the analyte can be reliably detected, while the LOQ represents the lowest concentration at which the analyte can be quantified with a satisfactory level of accuracy and precision. In this study, the signal-to-noise ratios of 3 : 1 and 10 : 1 were obtained for the LOD and LOQ, respectively. The calculated LOD for artemether was found to be 7.95 *μ*g/mL and that of lumefantrine was 0.064 *μ*g/mL. The LOQ was measured to be 24.10 *μ*g/mL for artemether and 0.193 *μ*g/mL for lumefantrine.  Table 3 shows the results of the LOD and LOQ of artemether and lumefantrine.

#### 3.2.5. Precision

Repeatability (intraday) studies showed a % RSD of 0.175 and 0.203 for artemether and lumefantrine, respectively. It was also observed that the intermediate studies (interday) showed a % RSD of 0.340 and 0.554 for artemether and lumefantrine, respectively. This implies that a change in days did not have a change in the precision of the developed method by more than 2% for both APIs. The results of the precision study for artemether and lumefantrine are presented in Table 4.

#### 3.2.6. Accuracy

The accuracy of the developed method is shown in Table 5. Based on the standard deviation (SD), relative standard deviation (% RSD) values, and the % drug recovery, the developed method is accurate at 3 levels of concentrations with % RSD in the range 0.004–0.072 for artemether and 0.092–0.511 for lumefantrine.

#### 3.2.7. Robustness

A slight modification of certain chromatographic conditions, including adjustments to the flow rate, column temperature, and mobile phase composition, resulted in a negligible variance of no more than 2% in the assay values, as illustrated in Table 6.

#### 3.2.8. Drug Assay of Market Samples (Quantitative Estimation of the API Content) Using the Validated Method

Assay analyses were performed on some selected brands of marketed drug products to investigate the content and concentration of the API present. The quantity of the two APIs was estimated using the calibration curve equation.  Table 7 presents the results of the analyzed market samples using the developed RP-HPLC method.

### 3.3. Greenness Study

The method development and validation processes were meticulously conducted in compliance with the principles of green chemistry and environmental friendliness (Table 8). This was achieved through the careful selection of chemicals for the mobile phase and the subsequent application of the developed method to analyze marketed samples.

## 4. Discussion

Numerous studies have endeavored to develop methodologies for quantifying active pharmaceutical ingredients (APIs) in fixed-dose combination drugs. However, challenges emerge in simultaneously determining artemether and lumefantrine in fixed-dose combination medicines. Given the difference in chemical properties of artemether and lumefantrine, classified as BCS class II or IV drugs with characteristics such as poor water solubility and limited permeability, a surge in research has been witnessed, aiming to establish methods for their distinct analysis in pharmaceutical and biological matrices. This approach persists due to the complexities of achieving optimal separation when both drugs coexist in a solution. The growing need for swift and cost-effective high-performance liquid chromatography (HPLC) methods is particularly pronounced in the resource-limited setting.

The investigation revealed the significance of the stationary phase selection, particularly concerning the separation efficiency of the drug. While the prevalent use of C18 (octadecyl) columns with acetonitrile as the main solvent is common, our study found that employing a C18 column resulted in prolonged analysis times and indistinctly separated peaks. Subsequently, utilizing an ODS Intersil-C8 column (Phenomenex) (150 × 4.6 mm with 5.0 *μ*m particles) as the stationary phase improved the peak shape and significantly reduced analysis time. This improvement is attributed to the shorter alkyl length of the C8 (octyl) column. These findings align with a method previously developed by Abd-AlGhafar et al. in 2022 [19]. They discovered that using the C8 column gave a shorter elution time and nicely separated peaks. Also, in a study where the Phenomenex Luna C18 column (150 × 4.6 mm with 5.0 *μ*m particles) was used as the stationary phase in the development and validation of an RP-HPLC method for simultaneous quantification of gefitinib and resveratrol, it resulted in short retention times of 2.56 minutes and 1.80 minutes, respectively [20].

The study employed a mobile phase composed of a 70 : 30 (v/v) mixture of acetonitrile and 0.05% orthophosphoric acid. Acetonitrile, chosen for its versatile ability to dissolve both polar and nonpolar analytes, is widely utilized in high-performance liquid chromatography (HPLC) due to its high chromatographic performance [21] and availability in the high-purity HPLC grade [22]. In addition, it is compatible with various stationary phases. Orthophosphoric acid was selected for its UV transparency and efficacy in optimizing the separation of artemether and lumefantrine when the pH is adjusted to approximately 3-4. Similar to a study conducted by Patel et al., a filtrate compatibility study was conducted to ensure the selection of an optimal filter for obtaining a refined filtrate for subsequent analysis [23].

The validation of the RP-HPLC method for artemether and lumefantrine assay was meticulously performed, adhering to the stringent guidelines inspired by the International Council for Harmonisation of Technical Requirements for Pharmaceuticals for Human Use (ICH). This encompassed a thorough examination of specificity, linearity, accuracy, precision, sensitivity (LOD and LOQ), and robustness by the regulatory framework set forth by the European Medicines Agency (1994) and ICH (Q2B, 1996). System suitability testing, involving six replicate analyses at concentrations of 1100 *μ*g/mL for artemether and 600 *μ*g/mL for lumefantrine, elucidated a notably favorable system performance. The obtained results indicate favorable system performance. Under ideal circumstances, a chromatographic peak is expected to exhibit a Gaussian shape. However, if the migration and distribution of the drug within the HPLC column are nonuniform, an asymmetric peak is obtained [24]. When the symmetry factor approaches 1, it suggests that the peak shape is approaching a desirable Gaussian distribution [20]. Tailing or fronting can occur when there is an uneven flow in the column. To mitigate tailing, various strategies can be employed, such as adjusting the pH or ionic strength of the mobile phase, optimizing the column temperature, using different stationary phases or additives in the mobile phase, or modifying the sample preparation techniques [24].

The method is specific, highlighting the method's ability to accurately identify artemether and lumefantrine, even in the presence of potential interferences. The linear regression analysis revealed exceptional linearity (*R*^2^ ≥ 0.99), a key indicator of the strong correlation between the signals obtained and the various levels of concentrations prepared. Determining LOD and LOQ values, using the signal-to-noise ratio (S/N) method, demonstrated the method's sensitivity in reliably detecting and quantifying artemether and lumefantrine irrespective of other impurities or excipients that might be present. The precision analysis, both intraday and interday, reveals a relative standard deviation (RSD) below 2%. This indicates that the method consistently yielded closely aligned results when the HPLC analysis was conducted within the same day or on different days under identical conditions. A method incapable of providing consistent results over multiple days suggests the potential for disparate values, rendering it unsuitable. Intentionally varying certain chromatographic conditions and parameters, the researchers assessed the method's robustness, and the % RSD value confirmed that the observed change was less than 2%, affirming its resilience and reliability in delivering expected results.

Comparing the proposed method with that developed by Habib et al. [25], the present method revealed better economic efficiency and shorter retention time, enhancing its practical utility in pharmaceutical analyses. Furthermore, the method's alignment with green chemistry principles, encompassing considerations of reagent toxicity, compatibility, energy consumption, and biodegradability, attested to its environmental conscientiousness.

In ensuring environmental friendliness, green chemistry plays a crucial role in method development. A greenness study was conducted, considering factors such as reagent toxicity, reagent compatibility, the energy consumption of electronic devices, biodegradability of reagents and other disposable materials, and the duration of the analysis. The results strongly indicate that the method development and validation process align very well with the principles of green chemistry.

## 5. Conclusion

A new high-performance liquid chromatography (HPLC) method has been developed and validated for the identification and quantification of artemether and lumefantrine in pharmaceutical dosage forms. This method offers a reliable and efficient approach to simultaneously analyze these compounds, saving time, eliminating the costs associated with separate analyses, and being very friendly to the environment. The method was validated for specificity, precision, accuracy, and robustness according to the International Council for Harmonisation of Technical Requirements for Pharmaceuticals for Human Use (ICH) guidelines. The developed analytical method proved satisfactory as per ICH guidelines and hence can be used for the determination and quantification of artemether and lumefantrine in bulk drug and pharmaceutical dosage forms during manufacturing or routine pharmacovigilance [12].

## Figures and Tables

**Table 1:** System suitability data for artemether and lumefantrine.

API	Injection	Retention time/min	Peak area/mAU	Symmetry factor	Theoretical plates
Artemether	1	5.38	250.243	0.91	2305
	2	5.38	249.452	0.90	2289
	3	5.37	251.245	0.91	2325
	4	5.38	250.365	0.91	2297
	5	5.37	250.054	0.90	2287
	6	5.38	251.144	0.91	2280
	Mean	5.377	250.417	0.91	2297.17
	SD	0.0047	0.6205	0.0052	16.1297
	% RSD	0.088	0.248	0.570	0.702

Lumefantrine	1	1.42	9466.254	1.31	5078
	2	1.42	9457.814	1.32	5019
	3	1.41	9472.300	1.31	4987
	4	1.42	9454.223	1.32	5100
	5	1.42	9447.235	1.32	5018
	6	1.42	9454.890	1.32	5009
	Mean	1.418	9458.786	1.32	5035.167
	SD	0.004	9.0418	0.0052	43.8151
	% RSD	0.29	0.10	0.39	0.87

Acceptance criteria: RSD ≤ 2%, tailing factor ≤2, theoretical plates ≥2000, and resolution (*R*) ≥2.

**Table 2:** Linearity results for artemether and lumefantrine.

API	Concentration (*μ*g/mL)	Mean peak area (mAU^*∗*^s)	*R* ^2^
Artemether	275	62.42	0.9992
	550	125.70	
	660	150.43	
	880	202.91	
	1100	241.94	
	1320	304.57	
	1540	351.16	
	1650	378.57	
	1925	441.59	

Lumefantrine	150	2385.89	0.9985
	300	4820.10	
	360	5721.26	
	480	7787.03	
	600	9461.54	
	720	11774.6	
	840	13336.3	
	900	14236.7	
	1050	16270.3	

**Table 3:** Limit of detection (LOD) and limit of quantification (LOQ).

Parameters	Artemether	Lumefantrine
Slope (s)	0.2296	15.609
SD	0.5534	0.3019
LOD (*μ*g/mL)	7.95	0.064
LOQ (*μ*g/mL)	24.10	0.193

**Table 4:** Precision study data for artemether and lumefantrine: repeatability and intermediate precision.

Concentration (*μ*g/mL) at 100%	Repeatability	Intermediate precision
	Peak area (mAU.s)	Peak area (mAU.s)
*Lumefantrine*		
	9465.165	9474.324
1100	9476.799	9501.247
1100	9422.657	9482.271
1100	9450.740	9356.301
1100	9456.740	9468.233
1100	9439.310	9430.342
Mean	9451.9018	9452.1197
SD	19.153865	52.403728
% RSD	0.20	0.55

*Artemether*		
600	252.641	253.100
600	252.541	252.341
600	252.642	252.603
600	252.583	251.712
600	251.524	250.789
600	252.166	251.342
Mean	252.3495	251.981
SD	0.44200577	0.8568
% RSD	0.18	0.34

Acceptance criteria: RSD ≤ 2%.

**Table 5:** Accuracy study.

API	Accuracy level (%)	Amount added (*μ*g/mL)	Amount recovered (*μ*g/mL)	% recovery	Mean % recovery	% RSD
Artemether	80	7945	7955	100.13	99.79	0.01
		8123	8098	99.69		
		8057	8022	99.57		
	100	10039	10032	99.93	99.93	0.00
		10041	10039	99.98		
		10043	10032	99.89		
	120	11950	12054	100.87	100.16	0.01
		12073	12045	99.77		
		12004	11985	99.84		

Lumefantrine	80	9709	9695	99.86	99.50	0.51
		9692	9587	98.92		
		9714	9688	99.73		
	100	12143	12062	99.33	99.33	0.22
		12140	12098	99.65		
		12133	12103	99.75		
	120	14556	14401	98.94	99.04	0.09
		14553	14423	99.11		
		14556	14421	99.07		

Acceptance criteria: RSD ≤ 2%.

**Table 6:** Results of the robustness study.

Condition	Modification	Mean area ± SD	Mean percentage content (%)	% RSD
Flow rate (mL/min)	0.90 (−1 mL/min)	248.25 ± 0.54	98.90	0.22
	1.10 (+1 mL/min)	252.38 ± 0.71	100.53	0.28

Column temperature (°C)	22 (−3°C)	250.05 ± 0.83	99.61	0.33
	30 (+5°C)	247.17 ± 0.09	98.47	0.04

Mobile phase composition (ACN: (0.05%) OPA)	60 : 40	249.36 ± 0.07	99.33	0.03
	65 : 35	248.18 ± 0.14	98.87	0.06

ACN: acetonitrile. OPA: orthophosphoric acid. Acceptance criteria: RSD ≤ 2%.

**Table 7:** Results of analyses of market samples using the developed RP-HPLC method.

Formulation	Sample code	Retention time L/A (min)	^*∗*^Percentage content of artemether (%)±SD	^Ψ^Percentage content of lumefantrine (%)±SD	Remarks on API content
Tablet	LZT	1.44/5.38	97.76±1.03	91.81±1.14	Passed
	LNT	1.41/5.38	99.68±0.89	97.55±1.05	Passed
	MLT	1.39/5.36	99.50±0.23	98.33±1.23	Passed
	LFT	1.40/5.38	99.49±0.18	96.83±0.28	Passed
	CTT	1.41/5.37	99.89±0.76	99.12±0.97	Passed
	TMT	1.39/5.38	96.90±0.25	91.64±1.11	Passed
	IDT	1.40/5.38	94.94±0.06	92.16±1.26	Passed
	DMT	1.39/5.38	99.68±0.34	96.52±0.73	Passed

Suspension	MLS	1.42/5.30	98.37±0.54	99.71±0.27	Passed
	LFS	1.42/5.40	99.92±0.32	97.98±1.09	Passed
	IDS	1.42/5.40	100.10±0.25	91.27±1.16	Passed
	LNS	1.42/5.43	98.50±0.88	97.72±1.02	Passed
	STS	1.42/5.42	116.76±0.94	80.35±1.01	Failed
	BMS	1.42/5.43	88.31±0.44	82.08±0.98	Failed

*L* = lumefantrine. *A* = artemether. ^*∗*^Acceptance criteria (BP): 90%–110%. ^Ψ^Acceptance criteria (BP): 90%–110%.

**Table 8:** Results of the greenness study.

Greenness study components	Factors considered	Strategies employed	^*∗*^Measure of success for greenness study on a scale of 0–10
Solvent selection	Lower toxicity renewable resources	Low-toxic solvents were used (acetonitrile and orthophosphoric acid)	8
Less solvent consumption	Utilize small quantities of solvents	A small volume (0.5 mL) of orthophosphoric acid was dissolved in 1000 mL of deionized water	9
Waste generation	Minimize waste effective use of resources	Required quantities were calculated before measuring to avoid waste	9
Energy consumption	Use of energy-efficient instrument	Low energy consumption (efficient) instruments were used in the study	8
Simple size and resource efficiency	Minimize sample size	A small sample size was utilized	9
Life cycle assessment	Assess the entire life cycle impact	Ensured a comprehensive understanding of the environmental impact	9
Biodegradability and environmental friendliness	Selection of biodegradable and environmentally friendly chemicals	All chemicals used were environmentally friendly and fast biodegradation	9
Compliance with green chemistry	Tests should comply with green chemistry principles	Green chemistry principles ensured	9
Reduction in analysis time	Short experimental/reaction time	Short run time in HPLC analyses. Use of a sonicator to speed up dissolution	9

^*∗*^(0): no success, (1–3): low success, (4–6): moderate success, (7-8): high success, and (9-10): very high success.

## Data Availability

The data used to support the findings of this study are included within the article. Raw data that support the findings of this study are available from the corresponding author upon request.

## Supplementary Materials

Supplementary Materials Supplementary Figures I and II illustrate the calibration curves of artemether and lumefantrine at 210 nm, respectively. These curves were used to establish the correlation between the concentration and the peak signals.
