# Supplementary material for: Development of the RP-HPLC Method for Simultaneous Determination and Quantification of Artemether and Lumefantrine in Fixed-Dose Combination Pharmaceutical Dosage Forms
Source: Adv Pharmacol Pharm Sci. 2024 Feb 7;2024:3212298. doi: 10.1155/2024/3212298 (PMC10866635; doi:10.1155/2024/3212298)
Supplement: Supplementary Materials — Supplementary Figures I and II illustrate the calibration curves of artemether and lumefantrine at 210 nm, respectively. These curves were used to establish the correlation between the concentration and the peak signals. [file 3212298.f1.docx]

**Supplementary Materials**

Figure I. Calibration curve of artemether at 210 nm

Figure II. Calibration curve of lumefantrine at 210 nm
